# Effect of POSS-Modified Montmorillonite on Thermal, Mechanical, and Electrical Properties of Unsaturated Polyester Nanocomposites

**DOI:** 10.3390/polym12092031

**Published:** 2020-09-07

**Authors:** Nidhin Divakaran, Manoj B. Kale, Duraisami Dhamodharan, Suhail Mubarak, Lixin Wu, Jianlei Wang

**Affiliations:** 1 CAS Key Laboratory of Design and Assembly of Functional Nanostructures, and Fujian Key Laboratory of Nanomaterials, Fujian Institute of Research on the Structure of Matter, Chinese Academy of Sciences, Fuzhou 350002, China; nidhin@fjirsm.ac.cn (N.D.); manojkale@fjirsm.ac.cn (M.B.K.); suhail@fjirsm.ac.cn (S.M.); 2 University of Chinese Academy of Sciences, Beijing 100049, China; 3 School of Mechanical Engineering, Xi’an Jiaotong University, Xi’an 710049, China; duraisamidhamodharan@fjirsm.ac.cn

**Keywords:** montmorillonite, aminopropyl isobutyl POSS, unsaturated polyester, mechanical properties, electrical conductivity, shrinkage weight loss

## Abstract

Montmorillonite (MMT) displays excellent cohesion with an unsaturated polyester (UP) matrix to generate a material which exhibits an extensive range of commercial applications. The organic modification of MMT using polyhedral oligomeric silsesquioxanes (POSS) and the effect of POSS-MMT on the thermal, mechanical, and electrical properties of UP are reported here. Transmission electron microscopy (TEM) images were used to characterize the modification of MMT using POSS. Modified MMT (POSS-MMT) was incorporated, at different wt.% (0.5, 1, 3, 5), into UP via in-situ polymerization. The presence of POSS-MMT enhanced the characteristic properties of UP as a consequence of good dispersion in the polymer matrix. Scanning electron microscopy (SEM) images support effective POSS-MMT dispersion leading to tensile strength enhancement of a UP/POSS-MMT nanocomposite (3 wt.% POSS-MMT) by 54.7% as compared to that for unmodified UP. TGA displays a 35 °C improvement of thermal stability (10% mass loss) at 5% POSS-MMT incorporation, while the electrical conductivity is improved by 10^8^ S/m (3 wt.% POSS-MMT) in comparison to that for unmodified UP. The conventional obstacle of UP associated with shrinkage weight loss during curing seems to be moderated with POSS-MMT incorporation (3%) resulting in a 27.8% reduction in shrinkage weight loss. These fabricated nanocomposites expand the versatility of UP as a high-performance material owing to enhancements of properties.

## 1. Introduction

The attractive influence of clay polymer nanocomposites has been quite imminent in recent years. Lots of research has been undertaken in inclining the blooming area of clay polymer technologies towards commercial applications [1,2,3]. Unsaturated polyester (UP) resins form the crux of thermosets capable to fabricate lightweight composites possessing admirable process ability, durability, and flexibility [4,5]. UP has a vital role in invigorating the tendency of clay polymer nanocomposites to be utilized as high-performance materials in an extensive range of products such as automobiles, construction and building materials, and electrical appliances [6,7]. The ample credit goes to the excellent reinforcement of nanoclay within UP, and several previous studies conclude this inference [8,9,10,11,12]. Montmorillonite (MMT) is one such form of nanoclay phyllosilicate mineral possessing one O–Al(Mg)–O octahedral sheet sandwiched between two O–Si–O tetrahedral sheets [13]. MMT-based polymer nanocomposites have been under constant scrutiny in recent years owing to its high aspect ratio, cation exchange capacity, superior surface area, and small particle size [14]. However, MMT reinforcement within a polymer matrix suffers certain impediments in the form of agglomeration which influences the dispersion and exfoliation of MMT in the polymer matrix. A prerequisite arises to organically modify the MMT (OMMT) prior to its interaction within the polymer matrix [15]. This arranges the affinity of MMT to exfoliate in the polymer with thorough dispersion. This information is crucial for hydrophobic polymers such as UP for a superior interaction with MMT. Several studies have been undertaken to analyze the property enhancement of UP with OMMT reinforcement [16,17,18,19]. The emphasis must be stressed on the appropriate choice of organic modifiers to exfoliate the MMT and evenly distribute in the UP matrix. The well-exfoliated OMMT provides impetus in the comprehensive property enhancement of UP.

Polyhedral oligomeric silsesquioxanes (POSS) have garnered a lot of attention recently due to their competence to boost the properties of polymer with exquisite reinforcement [20,21]. POSS structure comprises of a silica core as a backbone, with a Si atom attached on its vertex linking the O atom. The incorporated organic functional group attached to POSS moiety execute a vital role in modifying MMT and exfoliating the structure [22,23]. Recent reports have inferred the role of POSS as a nanofiller reinforcement in enhancing the thermal, mechanical, and electrical properties of the polymer [24,25,26,27,28,29]. POSS possess the affinity to organically attach with other nanofillers to achieve superior interaction with the UP matrix [30,31]. The contemplation towards hybrid amalgamation of POSS and MMT could relate to a meticulous interfacial interaction with the polymer matrix. Bi et al. explored this concept by incorporating POSS-MMT into poly(l-lactide) and obtained excellent improvement in thermal stability and gas permeation values [32]. Liu et al. surveyed the impact of POSS-modified MMT in the epoxy resin and enhanced its thermal stability [33]. Teo et al. examined the thermomechanical properties of anhydride-cured epoxy resin with reinforcement of POSS-imidazolium modified clay [34]. Zhao et al. explored the mechanical properties of POSS-MMT-incorporated polyamide 12 nanocomposites and achieved 60% amelioration of tensile modulus with 3% POSS-MMT [35]. However, no research has been specified to date on the effect of POSS-MMT on the UP matrix, which specifies the novelty of this research.

Our study focuses on the construction of a hierarchical network of POSS-MMT by organically modifying MMT using aminopropyl isobutyl POSS. Different wt.% of POSS-MMT are incorporated into UP via a solvent-free in-situ polymerization technique. The thermal, mechanical, and electrical properties of the as-prepared nanocomposites are evaluated to examine the influence of POSS-MMT in property enhancement. Another authoritative aspect during the fabrication of UP-based nanocomposites is the manifestation of shrinkage weight loss during the curing process due to free radical polymerization [36]. The effect of nanoclay in reducing the shrinkage weight loss of UP is predominant with previous studies supporting this hypothesis [37,38,39]. POSS, being a crystalline nanofiller, could be a potential candidate in assisting MMT in the process of limiting shrinkage weight loss [40]. The inclusive property improvement of the fabricated UP/POSS-MMT nanocomposites confers the role of a hybrid network of POSS and MMT in accomplishing interfacial reinforcement of UP. It validated the route towards the construction of UP-based nanocomposites with a facile polymer processing technique, minimum shrinkage weight loss, and minimal addition of nanofillers, thereby boosting the versatility of UP in industrial applications.

## 2. Experimental

### 2.1. Materials

Aminopropylisobutyl polyhedral oligomeric silsesquioxanes (POSS-NH_2_) were obtained from Passkey Technology Co., Ltd. (Changsha, China). Pristine montmorillonite was procured from the Sanding Technology Corporation (Zhejiang, China). Hydrochloric acid (HCl) 37% and tetrahydrofuran (THF) were supplied by Sigma Aldrich (St Louis, Mo, USA). Maleic anhydride (MA) and phthalic anhydride (PA) were purchased from the Aladdin Industrial Corporation (Shanghai, China). Propylene glycol (PG) and ethylene glycol (EG) were obtained from General Reagent (Shanghai, China). Diethylene glycol (DEG) and styrene were procured from the Xilong Corporation (Guangzhou, China). Cobalt iso-octanoate was purchased from the Shengfei Fine Chemical plant (Shanghai, China) and methyl ethyl ketone peroxide was procured from the AkzoNobel Corporation (Shanghai, China). Both are used as accelerator and free radical initiator, respectively. Hydroquinone was supplied by Aladdin chemicals (Shanghai, Country). All chemicals were utilized without any further purification.

### 2.2. Synthesis of POSS-Modified MMT (POSS-MMT)

Pristine clay (10 g) and distilled water (500 mL) were employed in a beaker and subjected to 4 h of stirring. POSS-NH_2_ (4 g) was dissolved in THF (10 mL) and 10% HCl (10 mL) was added into it and collectively stirred for 1 h. This POSS solution was mixed with the suspended clay and the solution was stirred overnight. The mixture was filtered and washed using deionized water to remove impurities and untreated POSS and was later dried in vacuum at room temperature.

### 2.3. Synthesis of UP Resin

The melt polycondensation technique was adopted to synthesize the pure UP resin [41]. A total of 16 g PG, 20.5 g EG, 32.5 g DEG, 72.5 g PA, and 27.5 g MA were collectively added in a three-neck flask with stirring equipment and heated progressively until the temperature reaches 120 °C. A total of 0.1 wt.% antioxidant (triphenyl phosphite) was interfused as an antioxidant into the mixture and the temperature was augmented to 160 °C with the complete melting of PA and SA. The polycondensation reaction instigated at 160 °C and conducted between 160 °C and 210 °C with the subsequent calculation of the acidic value. When the acidic value reached 70 mg KOH/g, the reaction was kept under vacuum at 210 °C until the acid value becomes lower than 26 mg KOH/g. The temperature was then brought down to 190 °C and 0.01 wt.% (to that of total resin) hydroquinone was added into the mixture. Styrene (35 wt.%) was mixed into the resin as the temperature approached 120 °C. The UP pre-polymer was subjected to free radical polymerization using cobalt iso-octanoate and MEKP to undergo the curing process. The resin was poured on the Teflon mold and the curing process was carried out for 3 h in room temperature and post-cured for 3 h at 80 °C in a vacuum oven.

### 2.4. Synthesis of UP/MMT-POSS Nanocomposites via In-Situ Polymerization

**Figure 1:**
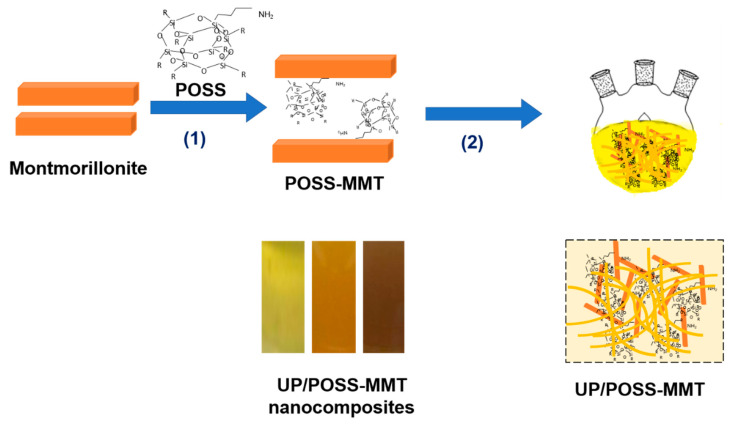
Schematic method to modify montmorillonite (MMT) using polyhedral oligomeric silsesquioxanes (POSS) and fabricate unsaturated polyester (UP)/POSS-MMT nanocomposites (**1**) functionalization with aminopropyl isobutyl POSS, (**2**) in-situ addition along with UP preparation after sonication with glycols. The inset contains images of nanocomposites with color variance from pure UP to 1% POSS-MMT to 5% POSS-MMT-added UP nanocomposites.

The various concentrations of POSS-MMT (0.5, 1, 3, and 5 wt.%) were added to the glycols used in the synthesis of UP resin, according to the scheme shown in Figure 1. The glycol mixture was subjected to ultrasonication for 1 h. The nano-dispersed POSS-MMT/glycol mixture along with other chemicals used in the UP-preparation procedure were constituted into a three-necked rounded beaker, with an overhead magnetic stirrer, and the whole polycondensation process for UP synthesis technique was adopted.

### 2.5. Characterization

X-ray diffraction XRD analysis was executed using Rigaku Miniflex600 (Tokyo, Japan) processed with Cu-Kα radiation (λ = 1.5406 Å) under the smaller angle of 2θ from 5° to 14°. Fourier Transform Infrared Spectra (FTIR) analysis was performed using a Perkin Elmer Spectrum One Spectrometer (Shelton, CT, USA) with KBr within the range of 500 to 4000 cm^−1^ under resolution of 4 cm^−1^. The Transmission Electron Microscopy (TEM) was carried out on JEM-2010 (JEOL, Tokyo, Japan) with an accelerating voltage of 200 kV. The samples were prepared by depositing a drop of solution on a copper grid with thin film formvar. Scanning Electron Microscopy (SEM) was performed using HITACHI, SU8010/EDX, Tokyo, Japan, with an energy-dispersive X-Ray spectrometer. Thermogravimetric analyses (TGA) were analyzed using TA Instruments STA449C (Newcastle, DE, USA) in the range of 25–800 °C under a nitrogen atmosphere with a heating rate of 10 °C/min. The tensile properties were analyzed using a universal testing machine (AGS-X PLUS, Shimadzu, Tokyo, Japan) according to ASTM D638 and ASTM D790 standards, respectively. The final values were calculated by taking the average values of a set of five specimens of each nanocomposite. Dynamic mechanical analysis (DMA) was performed on a TA DMA Q800 apparatus from TA Instruments (Newcastle, DE, USA). A single cantilever mode was used for testing. DMA tests were conducted from 30 °C to 180 °C with a heating rate of 3 °C/min at 1 Hz. Electrical conductivity measurement of the samples was executed using a four point or Kelvin probe method (Nanometrics, Milpitas, CA, USA). The specimens were cut to the size of 10 × 10 mm and coated on both sides with an electrodeposited silver paste (100 nm thick). The Ohmic contacts generated due to the silver paste aided in the measurement of the electrical conductivity in the Kelvin probe. The volumetric shrinkage was estimated using a facile technique of calculating the volumetric change of resins in the porcelain vials after curing. A known amount of resin was constituted in the porcelain vial and allowed to cure. The subsequent volume deficit in the resin (after curing) was compensated by adding some amount of water.

Volume of resin (before cured) = *V*_0_;

Volume of compensated water = *V*_w_;

Volume of resin (after cured) (*V*_1_) = *V*_0_ − *V*_w_;

Volumetric shrinkage (%) = $\frac{V_{1}\;}{V_{0}}$ × 100.

## 3. Results and Discussion

### 3.1. Structural and Morphological Analysis of POSS-Modified MMT

**Figure 2:**
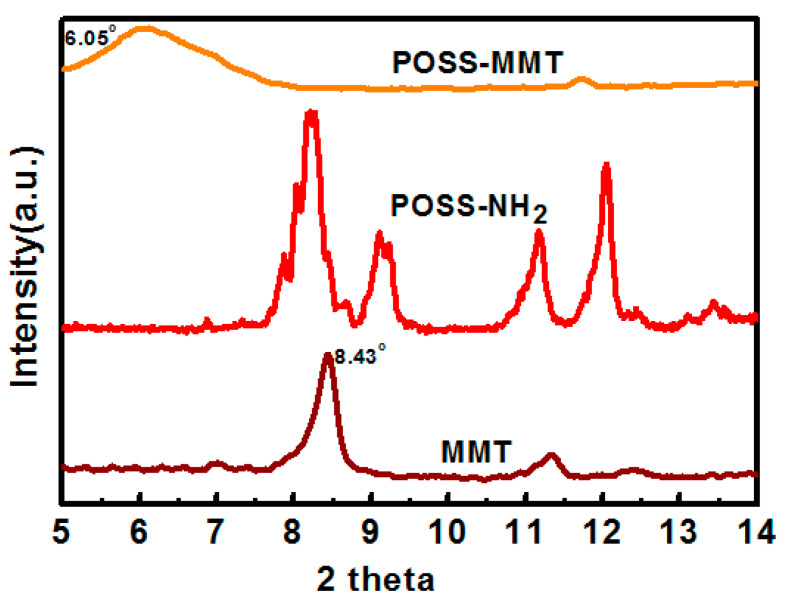
XRD inference for MMT, aminopropylisobutyl polyhedral oligomeric silsesquioxanes (POSS-NH_2_), and POSS-MMT.

The layered silicate MMT and its functionalization using POSS desires to be affirmed using the XRD analysis for the structural characterization. The immaculate modification of MMT using POSS was substantiated in this analysis. Figure 2 showcases the XRD patterns for MMT, POSS-NH_2_, and POSS-MMT. POSS-NH_2_ displays numerous peaks owing to its crystalline structure. The XRD diffraction peak of MMT attained at 2θ = 8.43° demonstrates the layered silicate structure of MMT, with the interlayer distance corresponding to 1.020 nm. The successful intercalation of POSS within the MMT could be vindicated in the XRD diffraction peak emerging at 2θ = 6.05°. The interlayer distance elevated to 1.4212 nm, computed using the Braggs law. The competence of MMT to interact with the surfactant POSS due to its cation exchange capacity prompted the intercalation of POSS within the layers of MMT [42]. The successful modification of MMT using POSS has a vital role in the reinforcement of the UP polymer matrix, devoid of agglomeration.

**Figure 3:**
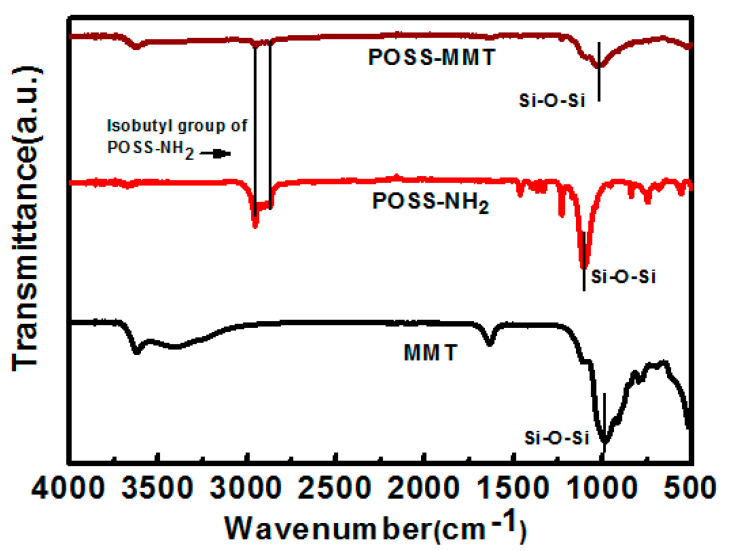
FTIR inference for MMT, POSS-NH_2_, and POSS-MMT.

Further evidence of organic modification of MMT using POSS could be obtained from the FTIR analysis. Figure 3 displays the FTIR transmittance spectra for MMT, POSS-NH_2_, and POSS-MMT, with each spectra citing a Si–O–Si bond around 1035 cm^−1^ [43]. The FTIR spectrum of pristine MMT exhibited a strong band at 3629 cm^−1^ due to the OH stretching vibration of structural OH groups [44]. POSS-NH_2_ FTIR spectra display a band at 1500 cm^−1^ attributed to the symmetric deformation of the NH_3_^+^ group. However, this group was not found in FTIR spectra of POSS-MMT due to overlapping with the strong bond of MMT, which substantiates the covalent bonding between pristine MMT and POSS [45]. The isobutyl groups of POSS-NH_2_ tend to be imbibed in POSS-MMT as interpreted in the FTIR spectra at 2869 cm^−1^. The vivid inference of successful intercalation of POSS within the layers of MMT could be obtained from this [46]. The FTIR spectra of POSS-MMT acquire the characteristics spectra of MMT and POSS-NH_2_, implying the attachment of POSS moiety amidst the layered MMT [47].

**Figure 4:**
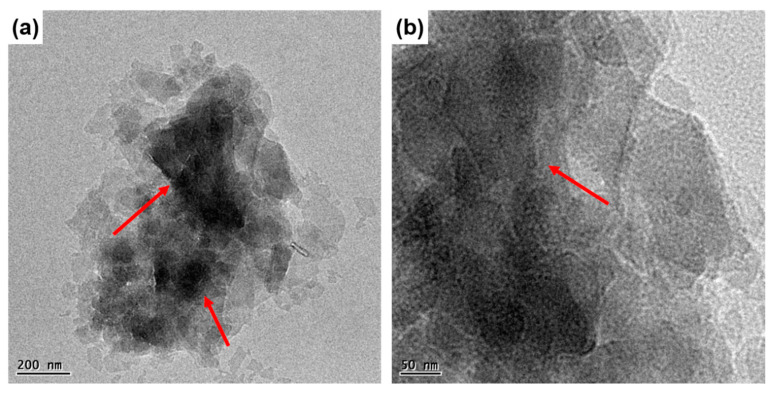
TEM images of (**a**) POSS-MMT; (**b**) higher magnification image of POSS-MMT.

The morphological aspects of POSS functionalized MMT is investigated by TEM analysis. Figure 4 furnishes the TEM images of POSS-MMT with different magnifications. The clay, as shown in Figure 4, displays minute tactoid morphology, which indicates that a majority of clay galleries have been exfoliated due to the covalent modification of POSS [48]. There are several dark hazy platelet-like structures on the surface of MMT. The platelets develop due to the presence of POSS which is responsible for the replacement of anions in MMT. However, there are a few microscopic inhomogeneities observed in the clay due to the presence of micron-sized intercalated clay aggregates. This is mainly due the inhomogeneous packing of POSS cations in POSS-MMT. There exists very strong POSS–POSS attraction forces allowing the diffusion of POSS molecules into the clay galleries. This has ultimately led to the exfoliation of MMT with the increase in their interlayer spacing [49]. The results are consistent with the XRD outputs.

### 3.2. Thermal Properties of UP/POSS-MMT Nanocomposites

**Figure 5:**
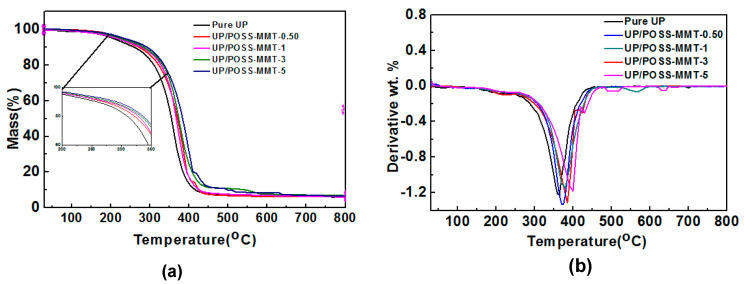
(**a**) TGA outputs of UP and UP/POSS-MMT nanocomposites with the inset demonstrating the enlarged view of thermal degradation (**b**) DTG plots of UP and UP/POSS-MMT nanocomposites.

TGA analysis was executed on UP/POSS-MMT nanocomposites to inspect the thermal stability of UP under different wt.% POSS-MMT reinforcement, as displayed in Figure 5a, and the results are expressed in tabular representation in Table 1. Figure 5b exhibits the DTG plots of UP and UP/POSS-MMT nanocomposites. The thermal degradation temperature of the nanocomposites tends to enhance compared to pure UP composites, owing to the superior interfacial reinforcement of UP with POSS-MMT. The initial decomposition observed in UP is due to the vaporization of physically absorbed water. The elevation in *T*_10_ values for UP/POSS-MMT nanocomposites demonstrates the delay in the onset of thermal degradation at 10% wt. loss. The *T*_10_ values of UP/POSS-MMT-3 is boosted by 35 °C, as compared to pure UP. This could be attributed to the presence of POSS-attached MMT in the polymer matrix which has relatively higher thermal stability [49]. The thermal degradation between 200 °C and 500 °C is ascribed to the decomposition of organic functional groups attached to POSS moiety. However, the presence of POSS has restricted the further degradation of UP at 50% wt. loss, as implied in Table 1, due to the molecular structure stiffness and high bond energy of Si–O in POSS [50]. The POSS-appended MMT has impeded the thermal decomposition of UP by posing as a barrier and confining the breaking of bonds of polymer. The credit infers to the excellent reinforcement of POSS-MMT within UP and this has led to the improvement in residual weight (%) in nanocomposites as compared to pure UP.

### 3.3. Morphological Properties of UP/POSS-MMT Nanocomposites

**Figure 6:**
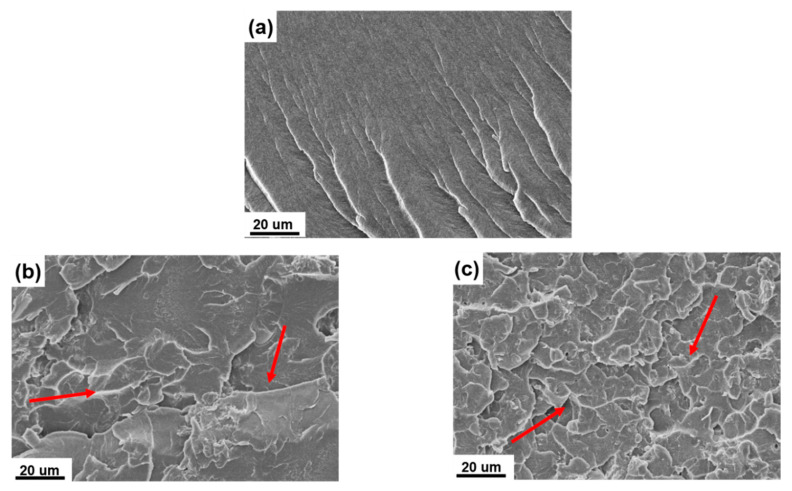
SEM images of (**a**) UP, (**b**) UP/ POSS-MMT-0.50, and (**c**) UP/POSS-MMT-3 nanocomposites. The red arrows in b represent the cracks developed due to stress load transfer over POSS-MMT in the UP matrix. The red arrow marks in c describe the homogenous dispersion of POSS-MMT and direction of crack propagation in the UP matrix.

The morphological analysis on the fractured surface of UP and UP/POSS-MMT nanocomposites was conducted and thoroughly examined using SEM to retrieve the vindication of a conventional outcome of possible reinforcement by POSS-MMT in UP. The SEM output of UP, as shown in Figure 6a, displayed a smooth surface with the least amount of cracks conceivably due to no addition of fillers. The SEM inference of nanocomposites (Figure 6b,c) exhibited several cracks in the fractured surface. The rationale lies in the generation of convoluted paths during crack propagation, as observed in Figure 6b,c (red arrows), in the fractured analysis [51]. POSS-MMT reinforcement hinders the crack propagation and forges the stress load transfer onto the polymer. This concept is coherent with the mechanical property enhancement of nanocomposites with addition of POSS-MMT. Figure 6b displays heterogeneous dispersion of POSS-MMT in UP, owing to the improper incorporation of POSS-MMT at 0.50 wt.%. The homogenous dispersion is visible in UP/POSS-MMT-3 (Figure 6c), providing the conjecture of immaculate incorporation of POSS-MMT [52]. The influence of POSS and MMT in intensifying the interfacial interaction of MMT with UP could be deduced from SEM outputs.

### 3.4. Mechanical Properties of UP/POSS-MMT Nanocomposites

**Figure 7:**
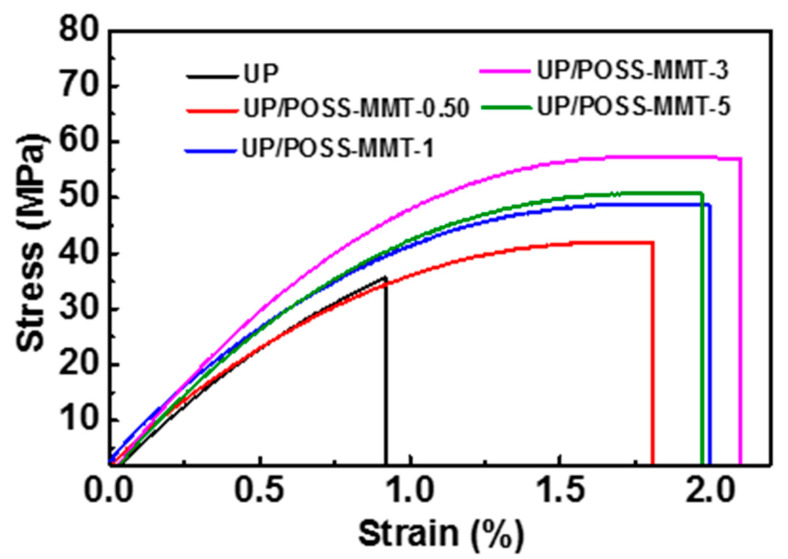
Stress–strain outputs for UP, UP/POSS-MMT nanocomposites.

The tensile strength analysis of the nanocomposites with different wt.% of POSS-MMT in UP are shown in Figure 7 and Table 2. The stress–strain curves in Figure 7 infer the considerable enhancement of tensile strength values of nanocomposites with the addition of POSS-MMT. The combined effect of POSS and MMT plays an important role in local stress concentration from the UP polymer to nanofillers. The meticulous interaction of POSS-MMT with UP matrix boosts the overall ductility of the polymer. The large surface area of POSS-MMT generates superior interfacial space for polymer/nanofiller interaction. The presence of POSS has enhanced the nanoscale dispersion of POSS-MMT into the matrix due to the strong covalent bonding between POSS and the polymer [53]. The octahedral structure of POSS represents a strong reinforcement factor in the polymer and acts as a crosslinking agent in UP. Table 2 interprets the tensile strength enhancement of UP with 3 wt.% POSS-MMT addition by 57%, as compared to pure UP. The reasoning transpires to be the potent stress load transfer mechanism from the polymer matrix to the nanofillers. However, the tensile strength of UP/POSS-MMT-5 appears to decline due to the agglomeration of nanofillers above the critical concentration. The intermittent dispersion at 5 wt.% POSS-MMT hampers the overall stress transfer mechanism, thereby dwindling the tensile strength. The elongation at break (%) values also imply a cumulative trend with POSS-MMT addition [54]. These values demonstrate the combined effect of POSS-MMT in enduring the stress applied on the polymer and ameliorating the mechanical properties.

**Figure 8:**
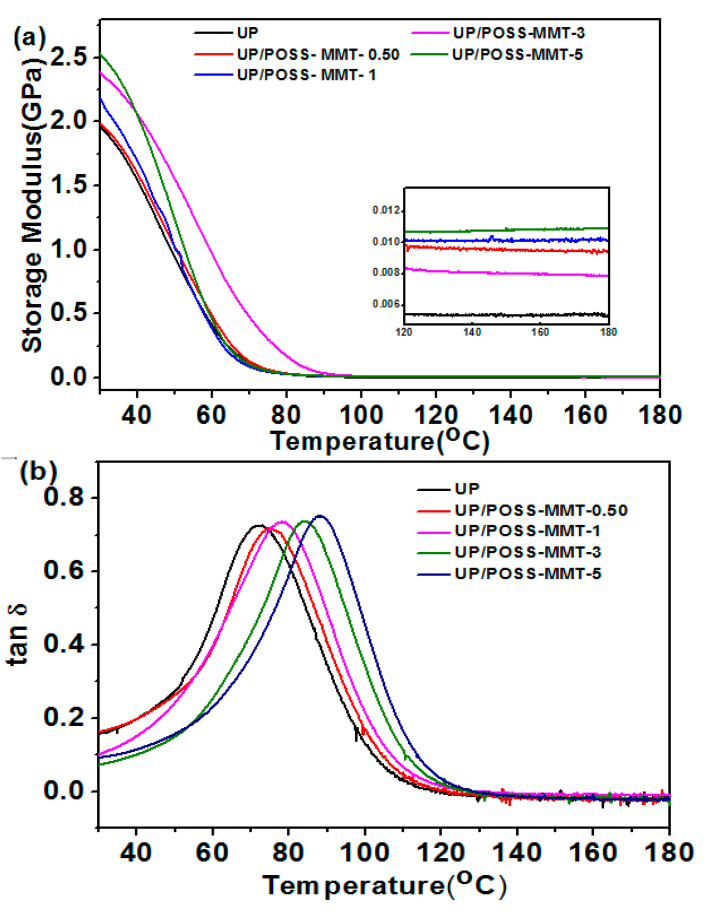
Dynamic mechanical analysis (DMA) inference for (**a**) storage modulus of UP, UP/POSS-MMT; and (**b**) tan *δ* representation for UP and UP/POSS-MMT. The inset in (**a**) depicts the storage modulus at high temperature (120 °C up to 180 °C) for the nanocomposites.

The thermomechanical properties of UP/POSS-MMT was analyzed using DMA, and their viscoelastic behavior was explored. The storage modulus values and tan delta plots of the nanocomposites are obtained from DMA analysis, as displayed in Figure 8 and Table 2. The combined effect of POSS and MMT manifested to be an excellent reinforcement agent within UP to enhance the storage modulus at 30 °C. The presence of POSS-attached MMT has improved the adhesion of nanofillers and the polymer matrix at the interface. Figure 8a displays the diminishing storage modulus of UP and their nanocomposites, with the increase in temperature. This cropped up due to the softening of polymer chains with temperature elevation. However, the crystalline POSS attached to MMT has deferred the softening phenomenon. Hence, we observe the augmentation of storage modulus values for different temperature intervals for the nanocomposites. The UP nanocomposite at 5% POSS-MMT addition exhibited 27.3% enhancement of the storage modulus, and this could be attributed to the superior interaction of the UP with POSS-MMT along with maximum stress load transfer from nanofillers onto the polymer matrix [55]. The glass temperature value (*T*_g_) of nanocomposites, derived from tan delta plots (Figure 8b), displays an increasing trend with the addition of POSS-MMT. The tan delta plots of nanocomposites also tend to maneuver in the right side, owing to the increase in crosslinking density of UP with POSS-MMT addition. The crystalline POSS has assisted MMT in accumulating molecular chain mobility within the polymer matrix [56]. This stemmed the perking up of *T*_g_ of nanocomposites, vindicating a strong interaction of the polymer–nanofiller interface. The unmitigated intercalation of MMT using POSS provided a strong influence on viscoelastic properties of nanocomposites.

### 3.5. Electrical Properties of UP/POSS-MMT Nanocomposites

**Figure 9:**
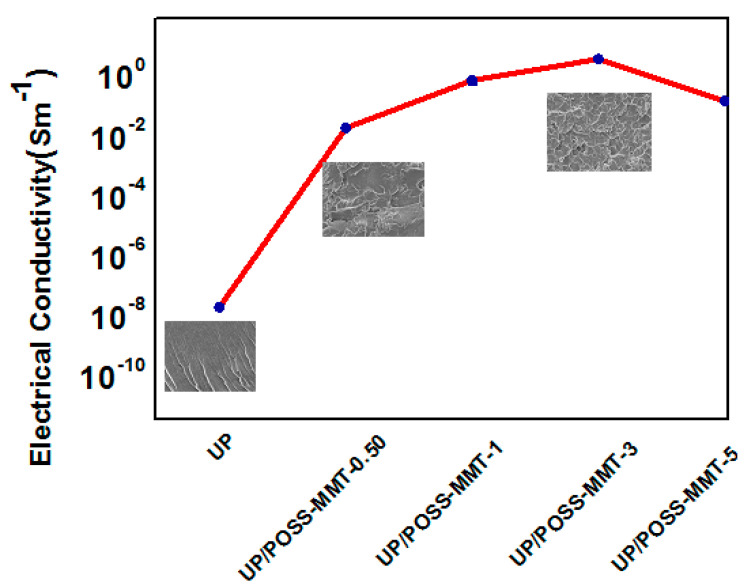
Electrical conductivity of UP and UP/POSS-MMT nanocomposites. The inset displays the SEM images (see Figure 6) which has correlation with conductivity amelioration, where the increase in wt.% of POSS-MMT has led to homogenous dispersion simultaneously enhancing electrical conductivity.

The interminable efforts to explore the overall aspects of nanocomposites to drive the polymer towards commercial application has prompted the electrical property study of UP/POSS-MMT nanocomposites. Figure 9 and Table 3 display increasing inclination of electrical conductivity values with different wt.% of POSS-MMT addition.

This rationalized the profound interaction of POSS-MMT with UP. The highly crystalline POSS plays an important role in exquisite interfacial interaction with polymer [57]. The superior aspect ratio of POSS-MMT constructs a manifold path for the electrons to flow, provoking a transcendent conducting network. This led to a boosting of electrical conductivity of UP/POSS-MMT nanocomposites with the technique of hopping, tunneling, and conduction in UP. The highly insulating UP gets a conductivity exhilaration with filling of insulating gaps by POSS-MMT [58]. The inset in Figure 9 vindicates this logic and has compelled for electrical conductivity enhancement by 10^8^ S/m for 3 wt.% POSS-MMT, with reference to pure UP. The SEM images (Figure 6c) surmise the conductivity enhancement concept with homogenous dispersion of POSS-MMT in the matrix. However, UP/POSS-MMT-5 demonstrates a steady decline in conductivity due to the agglomeration of POSS-MMT above the critical concentration [59]. The irregular dispersion of nanofillers has emanated the reducing of conductivity, nevertheless impacting the comprehensive conductivity of polymer. The uneven distribution of POSS-MMT at a higher concentration (5 wt.%) has mustered relentless contacts for the electrons to flow, thereby maintaining the polymer conductivity.

### 3.6. Shrinkage wt.% Loss of UP/POSS-MMT Nanocomposites

**Figure 10:**
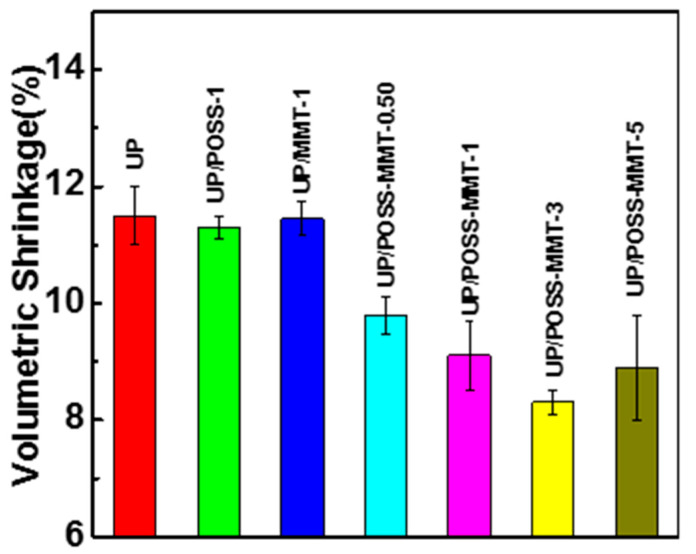
Volumetric shrinkage (%) of UP, UP/POSS, UP/MMT and UP/POSS-MMT nanocomposites.

The free radical polymerization initiated during the UP curing process impinge the overall weight of the polymer, resulting in shrinkage weight loss [60]. The onus on reducing the shrinkage weight loss relies on nanoclay approaching the polymer matrix and promoting the interfacial interaction. POSS-modified MMT impregnate the microstructure void within the UP matrix and that results in a reduction of shrinkage wt.% loss, as shown in Figure 10. However, UP with incorporation of 1% MMT and 1% POSS abstain from shrinkage weight loss reduction due to an improper interaction with UP. This explains the vitality of the synergistic effect of POSS-MMT in reducing the shrinkage phenomenon and furnishing interesting noteworthy outputs [36]. The homogenous dispersion of the nanofillers has yielded degradation in shrinkage wt. loss % from 11.5 (pure UP) to 8.3 at UP/POSS-MMT-3. The crystalline structure of POSS aided MMT for a compatible interaction with UP and concocting interfacial adhesion. The addition of POSS-attached nanoclay has limited the reaction induction time by posing as an auxiliary accelerator between UP and styrene during the curing process of UP in order to affect the shrinkage weight loss [39]. The agglomerated diffusion of POSS-MMT in UP at 5 wt.% perturbed the shrinkage weight loss with the values increasing from 8.3 to 8.9. This happens due to improper dispersion of POSS-MMT within the matrix. The impact of POSS-MMT in developing the polymer prevails low shrinkage weight loss and matures into a potential candidate for industrial applications.

## 4. Conclusions

The effect of POSS-MMT within the UP matrix was explored by fabricating UP/POSS-MMT nanocomposites via in-situ polymerization. POSS-MMT substantiated to be an excellent reinforcement in UP, owing to their superior aspect ratio and meticulous polymer interfacial interaction. This critical aspect steered towards inclusive property enhancement of UP. The thermal stability of UP elevated by 35 °C with 5 wt.% POSS-MMT addition. The combination of POSS and MMT posed as a barrier withstanding the thermal degradation of UP. The tensile strength of UP likewise demonstrated 54.7% amelioration with 3 wt.% POSS-MMT addition. This could be attributed to the admirable potential of POSS-MMT to achieve maximum stress load transfer to the polymer matrix. The electrical conductivity of UP/POSS-MMT also displayed 10^8^ S/m improvement due to the tendency of POSS-MMT to act as a conducting network in UP and overcoming the insulating characteristics of UP. The influence of POSS-MMT had an adverse effect on the volumetric shrinkage during the curing process of UP. The superb reinforcement characteristics of POSS-MMT in UP reduced the overall volumetric shrinkage of the polymer. Such high performance nanofillers unfurl the path of exploring the versatility of UP-based nanocomposites. The future scope of adapting the fabricated nanocomposites into fiber-reinforced plastics is imminent and this could explore the industrial application aspects of UP.

## Figures and Tables

**Table 1:** TGA measurement of UP/POSS-MMT nanocomposites.

Sample Name	*T*_10_ * (°C)	*T*_50_ * (°C)	Residual (%)
UP	258.4	356.2	6.34
UP/POSS-MMT-0.50	266.6	370.1	6.25
UP/POSS-MMT-1	279.7	371.3	6.40
UP/POSS-MMT-3	284.5	376.4	7.35
UP/POSS-MMT-5	295.4	381.3	6.98

* *T*_10_ and *T*_50_ represent the temperature during which 10% and 50% mass loss occurs.

**Table 2:** Mechanical and thermo-mechanical analysis of UP/POSS-MMT nanocomposites.

Sample Name	Tensile Strength (MPa)	Elongation at Break (%)	Storage Modulus at 30 °C (MPa)	*T*_g_ * (°C)
UP	35.2 ± 0.3	0.92 ± 0.015	1956	72.1
UP/POSS-MMT-0.05	41.9 ± 1.4	1.81 ± 0.001	1980	74.8
UP/POSS-MMT-1	48.6 ± 2.5	1.99 ± 0.023	2152	78.7
UP/POSS-MMT-3	56.8 ± 1.9	1.97 ± 0.030	2393	83.8
UP/POSS-MMT-5	50.2 ± 2.4	2.21 ± 0.100	2538	88.0

* *T*_g_ denotes the glass transition temperature.

**Table 3:** Electrical conductivity of UP/POSS-MMT nanocomposites.

Sample Name	Electrical Conductivity (S m^−1^)
UP	2.12 × 10^−8^ ± 0.0003
UP/POSS-MMT-0.50	1.96 × 10^−2^ ± 0.009
UP/POSS-MMT-1	0.7500 ± 0.030
UP/POSS-MMT-3	3.846 ± 0.135
UP/POSS-MMT-5	0.153 ± 0.025
